# Antibacterial efficacy of lytic phages against multidrug-resistant *Pseudomonas aeruginosa* infections in bacteraemia mice models

**DOI:** 10.1186/s12866-022-02603-0

**Published:** 2022-08-01

**Authors:** Shri Natrajan Arumugam, Prasanth Manohar, Sunilkumar Sukumaran, Sathish Sadagopan, Belinda Loh, Sebastian Leptihn, Ramesh Nachimuthu

**Affiliations:** 1 Anthem Biosciences Pvt Ltd., Bangalore, Karnataka India; 2 Antibiotic Resistance and Phage Therapy Laboratory, Vellore Institute of Technology, School of Biosciences and Technology, Vellore, Tamil Nadu 632014 India; 3 Zhejiang University-University of Edinburgh (ZJE) Institute, Zhejiang University, School of Medicine, Haining, 314400 Zhejiang, PR China; 4 Fraunhofer Institute for Cell Therapy and Immunology (IZI), Department of Antimicrobial Biotechnology, Leipzig, Germany; 5 Department of Infectious Diseases, Sir Run Run Shaw Hospital, Zhejiang University, School of Medicine, Hangzhou, PR China; 6 University of Edinburgh Medical School, Biomedical Sciences, College of Medicine & Veterinary Medicine, The University of Edinburgh, 1 George Square, Edinburgh, EH8 9JZ UK

**Keywords:** *Pseudomonas aeruginosa*, Antibiotic resistance, Phage therapy, Bloodstream infections, In vivo models, Phage efficacy

## Abstract

**Background:**

*Pseudomonas aeruginosa* is an opportunistic pathogen that can cause a variety of infections in humans, such as burn wound infections and infections of the lungs, the bloodstream and surgical site infections. Nosocomial spread is often concurrent with high degrees of antibiotic resistance. Such resistant strains are difficult to treat, and in some cases, even reserved antibiotics are ineffective. A particularly promising therapy to combat infections of resistant bacteria is the deployment of bacteriophages, known as phage therapy. In this work, we evaluated the in vivo efficacy of two *Pseudomonas* phages in bacteremia mice models. For this study, non-neutropenic mice (BalB/C) were infected with *P. aeruginosa* AB030 strain and treated using two bacteriophages, AP025 and AP006.

**Results:**

The results showed that a single dose of phages at higher concentrations, bacteria: phage at 1:10 and 1:100 were effective in eliminating the bloodstream infection and achieving 100% mice survival.

**Conclusion:**

This study highlights the efficacy of using a single dose of phages to restore mice from bacteremia.

**Supplementary Information:**

The online version contains supplementary material available at 10.1186/s12866-022-02603-0.

## Background

*P. aeruginosa* is one of the most common opportunistic pathogens that are known to cause nosocomial, acute and chronic infections [1]. *P. aeruginosa* is known to cause urinary tract infections, soft tissue infections, respiratory tract infections, gastrointestinal infections, bone and joint infections and many systemic infections. The diseases associated with this pathogen can lead to a high mortality rate, especially in patients with cystic fibrosis, cancer, burn wounds and immunocompromised patients [2, 3]. In recent years, treatment failure is mainly due to the increasing antibiotic resistance among clinical isolates. *P. aeruginosa* strains have been shown to have acquired resistance to a range of antibiotics including aminoglycosides and carbapenems, one of the last-resort antibiotics [4–6]. A recent report shows that the majority of healthcare-associated infections caused by *P. aeruginosa* are multidrug-resistant, leading to high numbers of deaths globally [7–9]. During the recent coronavirus pandemic, at least 30% of COVID-19 patients suffered from underlying bacterial infections, with *P. aeruginosa* having contributed to at least 12% of these infections [10, 11]. Most of the prophylactic treatment failure was due to antibiotic resistance or resistance which developed during the course of the prophylaxis [12, 13].

The increasing antibiotic resistance urgently calls for new non-antibiotic alternatives and researchers are looking at bacteriophages as one of the promising alternatives [14–16]. Phage therapy is the use of bacteriophages (phages) to treat bacterial infections. Recent studies and clinical outcomes suggest that phage therapy is a suitable alternative if antibiotic therapy fails due to drug resistance [17, 18]. Phages for therapy should be strictly virulent (also known as lytic), as studies have shown that prophages (genome-embedded phages with a lysogenic cycle) can alter the characteristics of a host and in some cases, increase virulence, not only in *P. aeruginosa* but also in other pathogens [19–21]. Many *Pseudomonas* phages with potential for clinical use have been reported [22–24], however, the majority of previously published studies on phage therapy focused mainly on wound infections [25, 26]. Our study describes the use of phages in bloodstream infections caused by the pathogen.

The two bacteriophages used in this study are *Pseudomonas* phages AP025 and AP006 which belong to the families, *Myoviridae* and *Siphoviridae*, respectively [27]. They were previously characterized for their in vitro activity against MDR and XDR strains of *P. aeruginosa* [27]. Here, AP025 and AP006 were used in mouse models to test their therapeutic value to treat infections in vivo.

## Results

### Phage therapy for bacteremia infections caused by *P. aeruginosa*

**Fig. 1:**
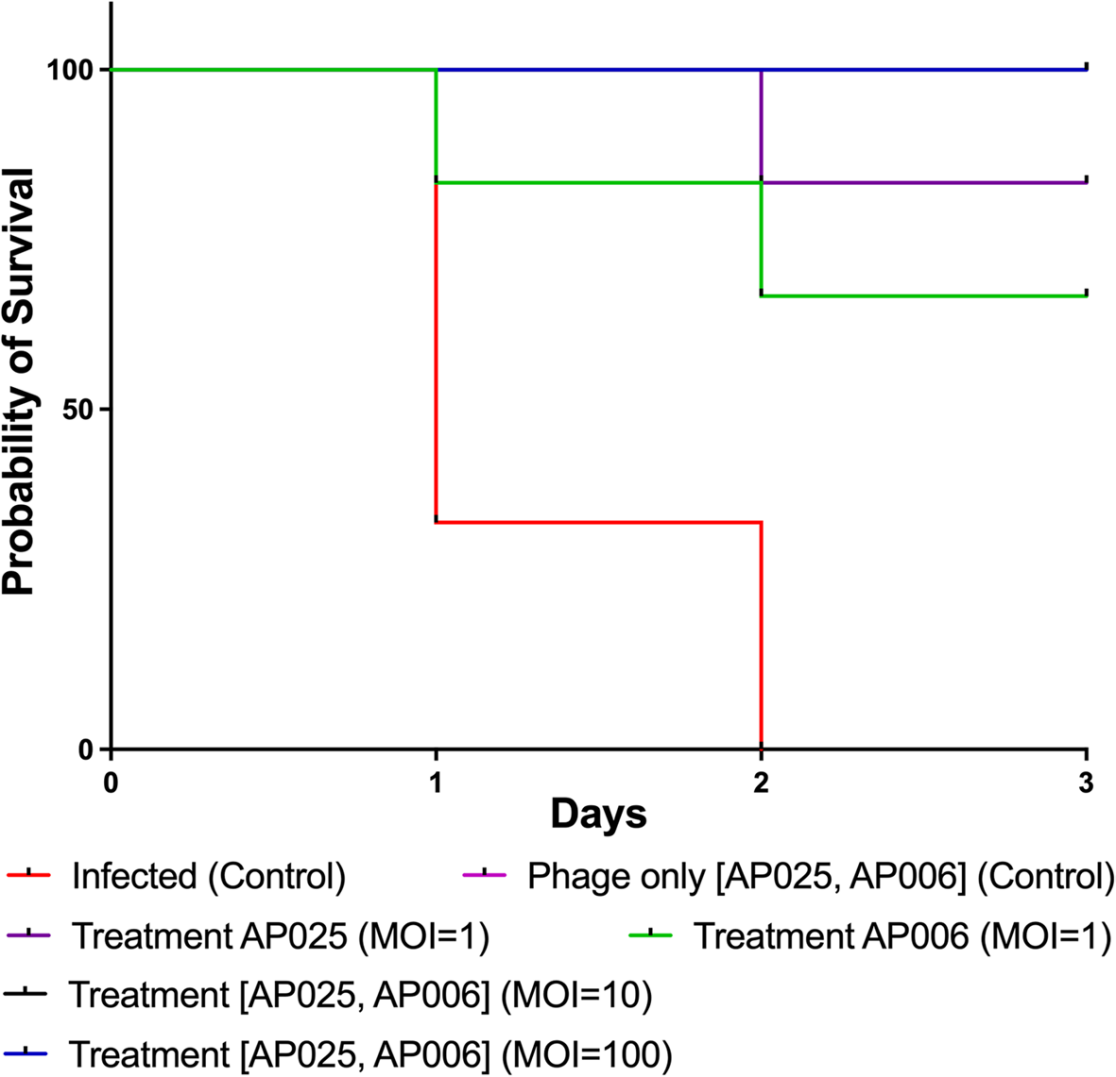
Pathogenicity of *Pseudomonas aeruginosa* AB030 in mice models and efficacy of *Pseudomonas* phage AP025 (**A**) and AP006 (**B**) against *P. aeruginosa* bacteremia infections. Representative survival curves of mice following infection by clinical *P. aeruginosa* strain AB030 and treated with phages 2 hours post-infection. The mice in the infected group were dead within 48 hours. In the phage control group, 100% survival was observed. When infected mice were treated with phage AP025 at MOI = 1, 1/6 mice were dead on the 2nd day. With phage AP006 at MOI = 1, we observed death in 2 of 6 mice on the 1st and the 2nd day. All the animals survived when treated with phages at MOI values of 10 and 100, respectively, with signs of infection. The survival curves were plotted using the Kaplan-Meier method and the log-rank test was used to analyze the difference in survival rates in GraphPad Prism 7.0. A statistically significant difference (*p* < 0.05) was observed in the treatment groups

When assessing the therapeutic value of phages for clinical use, animal experiments are necessary. Here, we used non-neutropenic BalB/C mice as an animal model for bloodstream infections. The mice were infected with *P. aeruginosa* via the intraperitoneal route (i.p.) which were also treated with *Pseudomonas*-specific phages and compared to the control groups. First, mice were injected with a dose of 8 × 10^6^ CFU of *P. aeruginosa* strain AB030 each via i.p. which would be lethal if untreated. Two hours post-infection, we administered one of the two phages that specifically infect AB030, phage AP025 or AP006. In the control group, the infected mice died within 2 days demonstrating that the bacterial dose was indeed lethal (Fig. 1). To show that the phage preparation had no negative effects on the mice, another control group was included. Here, mice were injected only with phages at MOI values of 1, 10 or 100 as single doses (i.p.). In this control group, we observed a 100% survival rate, indicating the absence of any toxic substance in the phage preparation. Using the identical dosing (or less), the phages were then used to test their effect on bacterial pathogens in a bacteremia mouse model.

The infected mice were treated with two phages, either AP025 or AP006 (Fig. 1). When treated with the phage AP025 at an MOI of 1 (8 × 10^6^ PFU/mice), five of the six mice survived (87.5%) with an observation time of 72 h. At an MOI of 10 (8 × 10^7^ PFU/mice) and MOI of 100 (8 × 10^8^ PFU/mice), 100% survival was observed and the animals showed no signs of infection. In the case where treatment was conducted with the AP006 phage at MOI 10 (8.4 × 10^7^ PFU) and 100 (8.4 × 10^8^ PFU), all the mice survived without any observable symptoms. Animals treated with the AP006 phage at MOI of 1 exhibited an 84% survival rate as 2 animals died, one after day 1 and the second after day 2 (Fig. 1).

### The effect of phage treatment on bacterial load in different organs

**Fig. 2:**
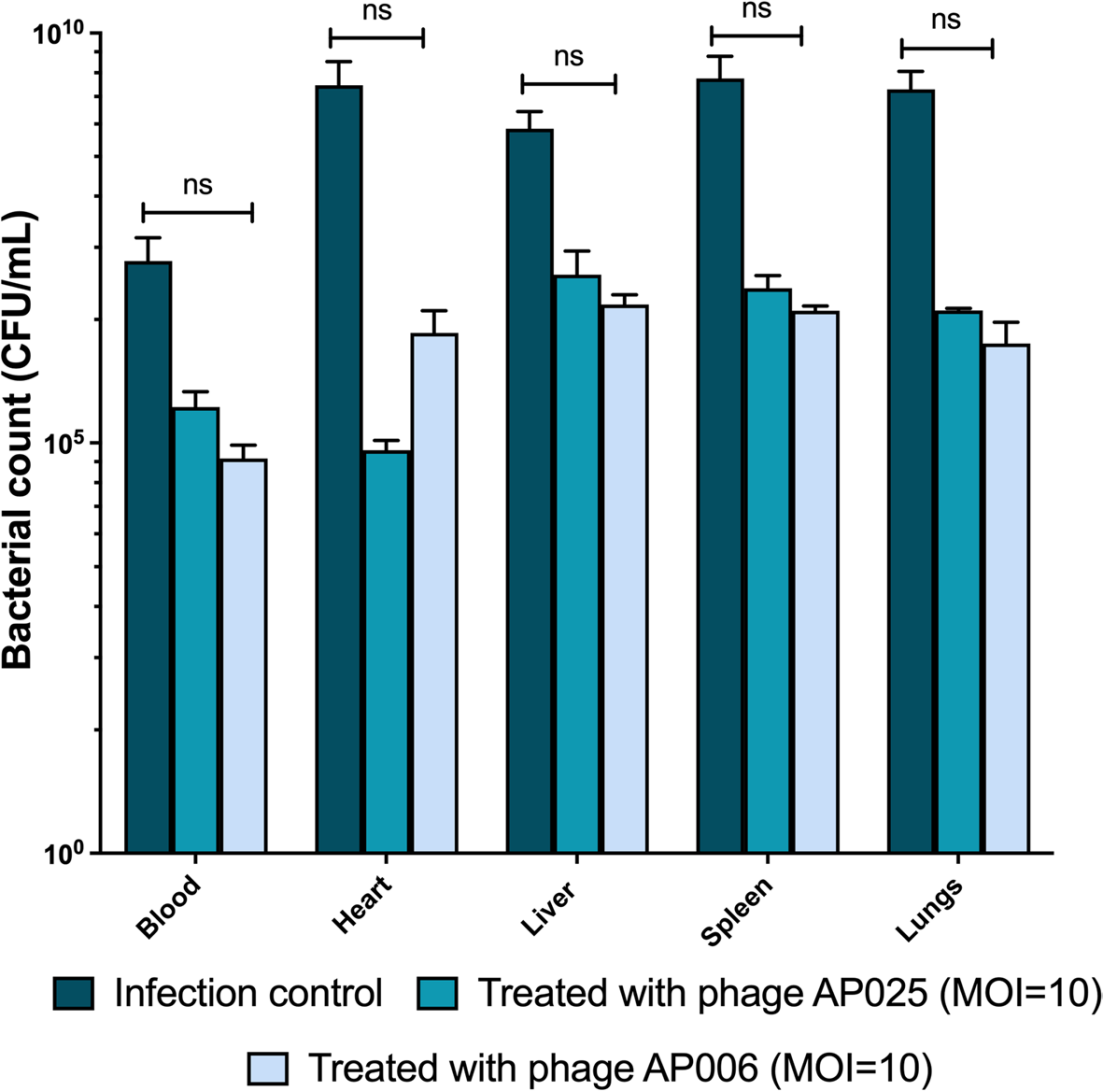
Bacterial enumeration from mice post-infection with *P. aeruginosa* and treated with *Pseudomonas* phage AB025 (MOI = 10) and AB006 (MOI = 10). Results are represented as CFU for bacteria isolated from different organs in the mice after 48 hours. Error bars represent the standard error of the mean (SEM) of three independent replicates. Data were analyzed using GraphPad Prism 7.0 and two-way ANOVA was used for statistical analysis (*P* < 0.05 was considered significant)

Phage therapy can be very efficient in clearing pathogens when the bacteria are planktonic, e.g. in the blood, while it can be more challenging if the bacteria are embedded within tissues or in a biofilm which might require encapsulation for optimized delivery [28]. Thus, we tested for bacteria in the infected and treated animals to determine how efficient the phages are at clearing the pathogen. Here, tissues were isolated from different anatomical regions or organs, quantified, homogenized and the colony-forming units of bacteria count. A substantial reduction in the bacterial count was observed in the phage treated groups compared to infection groups. After treatment, the liver, spleen and lungs contained the largest amount of bacteria, with 5.1 × 10^5^, 4.4 × 10^6^ and 4.1 × 10^6^ CFU/mL respectively. The number of bacteria was lower in the heart at 1.0 × 10^5^ CFU/mL, while the bacterial count in the blood was lowest at 1.2 × 10^4^ CFU/mL (Fig. 2).

## Discussion

*P. aeruginosa* is known to cause life-threatening infections, a situation that is aggravated by the high number of multidrug-resistant strains. Recent developments in phage research have demonstrated that phage therapy can facilitate the full recovery of infected mice in vivo studies [29–33]. Previously, we demonstrated that the *Pseudomonas* phages AP025 and AP006 were able to infect and lyse a wide range of multidrug-resistant *P. aeruginosa* strains [27]; however, they had not been tested in an in vivo model. While it is reasonable to assume that the phages might also be active in mammalian tissues, there are a plethora of unknown factors including the impact of the host immune system that requires vigorous testing of the phages in a mammalian model. To this end, we choose non-neutropenic BalB/C mice which were infected with *P. aeruginosa* via the i.p. route. Even equal amounts of phage to bacterial cells were able to show that the viruses were highly efficient in neutralizing the pathogen; at higher doses, treatment with either of the two phages allowed full recovery of the infected animals.

In our work, no phage-resistant mutants were observed during both, in vitro studies or in vivo treatments [34–37], indicating that these two phages are extremely efficient in infecting and killing the pathogen. This is even more astounding as a single dose of phage administration is effective. No negative effects caused by the phage preparations in mice clearly show that the phage preparations were free of bacterial toxins that have the potential to cause severe harm to the animals.

In the treatment groups, the animals were monitored for mortality, clinical symptoms and body weight for up to 72 hours post-infection. Infected mice which received the higher concentrations of phage, AP025 and AP006, were fully protected (100%) and showed no signs of infection. At lower concentrations of phages, i.e. with AP025 at a 1:1 ratio of phage: bacteria, five out of six mice survived (87.5%) for the duration of the experiment (72 h). Animals treated with AP006 phages at an MOI of 1 exhibited an 84% survival rate as 2 animals died. Previous studies on bacterial keratitis showed the dose-dependent clearance of the bacterial load, in which the MOI of 100 reduced the bacterial load by 99.78%. Similarly, previous studies also showed that phages at higher concentrations up to 10^7^–10^8^ PFU/mouse can substantially increase the survival percentage [29, 38]. Although phages are auto-dosing naturally due to their replicative nature if a suitable host is encountered, the number of phages administered will decide the therapeutic outcome which is not necessarily correlated with a multi-dosage regimen.

Our work is a proof-of-concept study which has limitations as the findings cannot be compared directly with a clinical therapy of a human patient: First, we investigated the infection and the efficacy of the phages within a short time frame after the inoculation with the pathogen (2 hours), before the onset of a complete infection. However, the animals in the control group (infected with bacteria, without phage) displayed several (partially severe) symptoms of infection 2 hours post-inoculation. Second, in our work, only a single dose of phage treatment was administered and not compared to two or more courses of phage injections. In clinical phage therapy, it is often necessary to administer multiple doses which are also occasionally given at lower phage concentrations.

When the infected and treated animals were tested for bacterial load, the liver, spleen and lungs contained the largest amount of bacteria and the number of bacteria was lower in the heart, while the bacterial count in the blood was the lowest. While it is not possible to determine where phages are most effective as we obtained fairly large deviations in phage numbers (expressed in our error bars in Fig. 2), we can observe tendencies where most bacteria can be found and how efficient our tested phages are compared to no phages. The efficacy of phages compared to each other is similar, showing the highest reduction in bacterial load in blood, compared to the absolute CFU numbers. Regardless of the variations in phage count, we could establish that the phages can reduce (48 hours) or abolish the bacterial infection, as after 72 hours, no viable bacteria were detected in the samples from animals treated with either phage (data not shown).

Phages are an alternative therapy to treat MDR infections but many questions remain about their use in systemic infections. This study showed the dose-dependent recovery from bacteremia in mice using a single dose of phages and the reduction of bacterial load after 48 hours post-treatment. This kind of study sheds light on the future of phage therapy and its therapeutic applications.

## Conclusion

In our short report, we demonstrate that the recovery of mice infected with the pathogen *P. aeruginosa* AB030 is significantly increased when two different therapeutic phages are administered. The current treatment for bacteremia caused by *P. aeruginosa* is the use of different antibiotics in varying combinations. However, for drug-resistant strains, phage therapy may not only be an alternative but possibly the only choice.

## Methods

### Bacterial strains and bacteriophage

*P. aeruginosa* isolate used in this study was collected from the super-speciality and tertiary care hospital in south India. *P. aeruginosa* strain AB030 was isolated from a patient with bacteremia. The bacteria were cultured in Luria-Bertani (LB) broth and the diluted (1:100, v/v) overnight culture at 10^6^ colony-forming units/mL (CFU/mL) was used for the experiments. From the previous studies, the isolate was found to be multi-drug resistant (MDR) and cause bacteremia in mice [16]. For the treatment, two previously characterized bacteriophages were used which include *Pseudomonas* phage AP025 and *Pseudomonas* phage AP006 [22]. The characterized *Pseudomonas* phage AP025 (*Myoviridae*) had infectivity of 39% and *Pseudomonas* phage AP006 (*Siphoviridae*) with 30% infectivity against 51 *P. aeruginosa* tested. Both bacteriophages AP025 and AP006 infect *P. aeruginosa* AB030 in in vitro experiments [27].

### Bacteriophage purification and assay

All the bacteriophages used in this study were precipitated using polyethylene glycol (PEG) and sodium chloride (NaCl). Briefly, 10% PEG and 1 M NaCl were added to phage lysate and incubated overnight at 4 °C. The precipitated phage particles were recovered by centrifugation at 12,000×g for 30 min and the supernatant was discarded. To the pellet, SM buffer was added and incubated at room temperature for 1 hr. To remove the cell debris from the phage suspension, an equal volume of chloroform was added and vortexed for 30 sec. The organic and aqueous phases were separated by centrifugation at 4000×g for 10 min and the aqueous phase containing phage particles was recovered and stored at − 20 °C. To test the infectivity of bacteriophages against *P. aeruginosa*, both spot test and double agar overlay methods were performed and plaque-forming units (PFU/mL) were calculated [27]. For the in vivo experiments, both phages were prepared at different multiplicity of infections (MOI) or phage to bacteria ratios at 1, 10 and 100. MOI can be defined as the number of phage particles against the number of bacteria.

### Phage therapy in the mouse infection model

For the in vivo studies, six-week-old BalB/C mice were used. The animals were housed at four mice per cage and maintained in standard laboratory conditions. The animals were raised and cared for by guidelines established by the Committee for the Purpose of Control and Supervision of Experiments on Animals (CPCSEA) in India. All procedures, care and handling of the animals were reviewed and approved by the Institutional Animal Ethical Committee (IAEC) of Anthem Bioscience.

#### Bacteremia model

To evaluate the efficacy of *Pseudomonas* phages in reducing the blood-stream infection, a total of 54 non-neutropenic mice (BalB/C) were chosen (Fig. S1). The clinical *P. aeruginosa* AB030 strain was used for bacteremia infection and two bacteriophages, AP025 and AP006 were used for treatment. Briefly, in the infection control group three mice were infected with *P. aeruginosa* AB030 (8 × 10^6^ CFU/mice) via the intraperitoneal (i.p.) route. In another infection control group, three mice received 100 μL of PBS. For the phage control group (*n* = 12), four groups of mice (3 per group) were injected with a single dose of AP025, AP006 (MOI = 10, 100) via i.p. In the treatment group or challenge studies (*n* = 6 mice/group), after 2 h of *Pseudomonas* infection a single dose of AP025 and AP006 (separately) phages were injected at MOIs of 1 (8 × 10^6^ PFU/mice), 10 (8 × 10^7^ PFU/mice) or 100 (8 × 10^8^ PFU/mice) (100 μL) via i.p. The mice were sacrificed (*n* = 3) immediately after death in the case of infection groups and after 48 hours in a treatment group (MOI of 10) and major organs i.e. blood, heart, liver, spleen and lungs were collected, homogenized, diluted, and evaluated for the bacterial load. Bacterial load was recorded as CFU/mL and the values were represented as standard errors of the mean (SEM).

### Statistical analysis

All the experiments were performed in duplicates and error bars were plotted using the standard errors of the mean (SEM). Significant differences were determined using two-way ANOVA with a *p*-value < 0.05. Survival curves were plotted using the Kaplan-Meier method, and the log-rank test was used to calculate the difference in survival rates using GraphPad Prism 9.0. Based on the log-rank test *p* < 0.05 was considered statistically significant.

## Supplementary Information

**Additional file 1: Figure S1.** Outline of the study groups used to evaluate the efficacy of two *Pseudomonas* phages in reducing the bacteremia in mice models.

## Data Availability

The dataset used and/or analysed during the current study is added in the manuscript. Additional datasets are not available with the authors.
