# Supplementary material for: Antibacterial efficacy of lytic phages against multidrug-resistant Pseudomonas aeruginosa infections in bacteraemia mice models
Source: BMC Microbiol. 2022 Aug 1;22:187. doi: 10.1186/s12866-022-02603-0 (PMC9340724; doi:10.1186/s12866-022-02603-0)
Supplement: Supplementary file 1 — Additional file 1: Figure S1. Outline of the study groups used to evaluate the efficacy of two Pseudomonas phages in reducing the bacteremia in mice models. [file 12866_2022_2603_MOESM1_ESM.docx]

**Supplementary material:**

**Figure S1: Outline of the study groups used to evaluate the efficacy of two *Pseudomonas* phages in reducing the bacteremia in mice models.**

**Bacteremia infection model**

**Treatment group**

**Infection control group**

**Phage control group**

N=3 mice, infected with MDR *P. aeruginosa* AB030 (8x10^6^ CFU/mice)

N=3 mice, injected with phage APφ006 (MOI=10) via IP.

N=3 mice, injected with phage APφ025 (MOI=10) via IP.

N=3 mice, injected with 100 µL of PBS via IP.

N=3 mice, injected with phage APφ006 (MOI=100) via IP.

N=3 mice, injected with phage APφ025 (MOI=100 µL) via IP.

N=6 mice, infected with MDR *P. aeruginosa* AB030 (8x10^6^ CFU/mice) and phage APφ006 (MOI=1) via IP.

N=6 mice, infected with MDR *P. aeruginosa* AB030 (8x10^6^ CFU/mice) and phage APφ025 (MOI=1) via IP.

N=6 mice, infected with MDR *P. aeruginosa* AB030 (8x10^6^ CFU/mice) and phage APφ025 (MOI=10) via IP.

N=6 mice, infected with MDR *P. aeruginosa* AB030 (8x10^6^ CFU/mice) and phage APφ006 (MOI=10) via IP.

N=6 mice, infected with MDR *P. aeruginosa* AB030 (8x10^6^ CFU/mice) and phage APφ006 (MOI=100) via IP.

N=6 mice, infected with MDR *P. aeruginosa* AB030 (8x10^6^ CFU/mice) and phage APφ025 (MOI=100) via IP.
